# Draft Genome Sequence of the Yeast Vanrija humicola (Formerly Cryptococcus humicola) Strain UJ1, a Producer of d-Aspartate Oxidase

**DOI:** 10.1128/genomeA.00068-18

**Published:** 2018-03-15

**Authors:** Daiki Imanishi, Katsumasa Abe, Yoshio Kera, Shouji Takahashi

**Affiliations:** a Department of Bioengineering, Nagaoka University of Technology, Nagaoka, Niigata, Japan

## Abstract

Vanrija humicola (Cryptococcus humicola) strain UJ1 is a basidiomycetous yeast that produces d-aspartate oxidase, which is highly specific to d-aspartate. Here, we report the 22.6-Mb draft genome sequence of V. humicola strain UJ1, which comprises 22.6 Mb in 46 scaffolds, with an overall G+C content of 62.82%, comprising 46 scaffolds with an *N*_50_ of 1.34 Mb.

## GENOME ANNOUNCEMENT

Vanrija humicola strain UJ1 (=JCM 9575), formerly known as Cryptococcus humicola strain UJ1, is a basidiomycetous yeast that utilizes d-aspartate as a sole source of carbon, nitrogen, or both, which is caused by a flavin enzyme, d-aspartate oxidase (DDO) (1, 2). DDO of V. humicola UJ1 is produced only in the presence of d-aspartate in culture media and has higher catalytic activity and specificity toward d-aspartate than DDOs from other origins (1, 3), which makes it useful for d-aspartate identification and quantification. The yeast draft genome sequence provides information on the d-aspartate-specific induction mechanism and the physiological significance of DDO in the yeast.

The genome sequence of V. humicola strain UJ1 was generated using an Illumina HiSeq 2500 platform. The sequencing generated 44,746,782 paired ends that were used for *de novo* assembly with Velvet version 1.2.10 (4) and Platanus version 1.2.4 (5) software. This assembly represents a total of 46 scaffolds with total and average lengths of 22,628,423 and 491,922 bp, respectively. The maximum and minimum scaffold lengths were 3,532,612 and 151 bp, respectively. The scaffold *N*_50_ and *N*_90_ values were 1,340,400 and 602,907 bp, respectively. The overall G+C content was determined to be 62.82%. Gene prediction using AUGUSTUS (6) trained with the parameters of the species Cryptococcus neoformans resulted in 8,919 genes and 37,033 exons. Additionally, 320 tRNAs were predicted using tRNAscan-SE (7). An automatic annotation of predicted open reading frames was carried out using Blast2GO Basic (8).

### Accession number(s).

This whole-genome shotgun sequencing project has been deposited in DDBJ/EMBL/GenBank under the accession numbers BFAH01000001 to BFAH01000046 (scaffolds 1 to 19 and 21 to 47, respectively). This paper describes the first version of the genome.
